# Protein expression profile of *Gasterophilus intestinalis *larvae causing horse gastric myiasis and characterization of horse immune reaction

**DOI:** 10.1186/1756-3305-2-6

**Published:** 2009-01-08

**Authors:** Liselore Roelfstra, Cornelia A Deeg, Stefanie M Hauck, Christina Buse, Mathieu Membrez, Bruno Betschart, Kurt Pfister

**Affiliations:** 1 Institute of Biology, University of Neuchâtel, Switzerland; 2 Helmholtz Zentrum München, German Research Center for Environmental Health (GmbH), Neuherberg, Germany; 3 Institute of Animal Physiology, Ludwig Maximilians University, Munich, Germany; 4 Institute of Tropical Medicine and Parasitology, Ludwig Maximilians University, Munich, Germany

## Abstract

**Background:**

Little information is available on the immunological aspect of parasitic *Gasterophilus intestinalis *(Diptera, Oestridae) larvae causing horse gastric myiasis. The objectives of this research were to analyze the protein content of larval crude extracts of the migrating second and third larvae (L2 and L3) of *G. intestinalis *in order to characterize the immune response of horses.

**Results:**

The proteomic profile of L2 and L3, investigated by using one and two dimensional approaches, revealed a migration pattern specific to each larval stage. Furthermore, Western blots were performed with horse sera and with sera of Balb/c mice immunised with the larval crude extracts of L2 or L3, revealing a different immune reaction in naturally infected horses *vs*. artificially induced immune reaction in mice. The comparisons of the immunoblot profiles demonstrate that the stage L2 is more immunogenic than the stage L3 most likely as an effect of the highest enzymatic production of L2 while migrating through the host tissues. Fifteen proteins were identified by mass spectrometry.

**Conclusion:**

This work provides further information into the understanding of the interaction between *G. intestinalis *and their host and by contributing a novel scheme of the proteomic profile of the main larval stages.

## Background

Nine species of *Gasterophilus *(Diptera, Oestridae) flies have been described causing, in the larval stage, gastrointestinal myiasis in equids. While *Gasterophilus intestinalis *(De Geer, 1776) and *Gasterophilus nasalis *(Linnaeus, 1758) are distributed worldwide and are often the only species reported in many parts of the New World, the remaining species are only reported in very limited areas of Europe, Eastern Countries [1] and Africa [2]. Adult bot flies deposit their eggs on the hosts' hair at different locations depending on the species of *Gasterophilus *[3]. *G. pecorum *is an exception as females lay their eggs on grass, leaves and stems of plants [1]. Infection occurs when eggs are introduced into horse mouth by animal licking and grooming. The first larval stage (L1) hatches, starts migrating and moulting into the second larval stage (L2) in the oral cavity [4]. Larvae of different species of *Gasterophilus *are specifically present in one or more regions of the gastrointestinal tract where the third larval stage (L3) remains attached to the mucosa for about 8–10 months [5]. The clinical signs associated with the migration and maturation stages of the larvae are difficult to diagnose, but it has been shown that different species of *Gasterophilus *can cause severe damages during their life cycle [6-9].

In the past years studies concerning the immunology and immunopathology of many oestrid myiasis causing larvae have increased because of their important implications in diagnostics and in immunisation programmes [10]. While immunological studies were mainly focused on *Hypoderma *cattle grub infection [11], and sheep nasal oestrosis by *Oestrus ovis *[12], the immunology of *Gasterophilus *spp. caused myiasis received little attention. This is also due to the inherent difficulties in studying immunological host-parasite interactions at the gastrointestinal mucosa interface. As a consequence, so far no major immunogens have been reported [13]. A single study discussed the development of antibodies for the diagnosis of myiasis by *G. intestinalis *larvae although the specificity of the immune reaction was not tested in the occurrence of concomitant horse parasitic infection [14]. More recently, many proteomics-based analyses, combined with two-dimensional gel-electrophoresis, have offered a comprehensive approach to better understand biological and immunological processes of pathogens and diseases [15-17]. The aim of this study was to characterize L2 and L3 proteins of *G. intestinalis *and to analyze the immune response of horses and immunized mice against larval antigens.

## Results

### 1-D analysis of the larval crude extract (LCE) of L2 and L3

**Figure 1:**
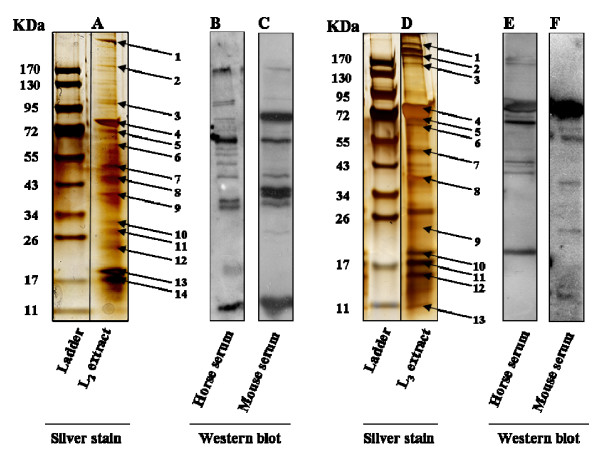
**1-D analysis of the LCE of L2 and L3**. Representative 1-D silver-stained gel of LCE of L2 (A) and L3 (D). The arrows indicate the bands that were selected for mass spectrometry. Western blot analysis of L2 incubated with horse serum (B) and mouse serum (C). Western blot analysis of L3 incubated with horse serum (E) and mouse serum (F). The protein identification by MS is presented in Table 1 and 2.

Migration of the LCE2 on the 1-D silver-stained gel showed a specific pattern (Figure 1A) with 14 bands that were isolated from the gel for further identification by mass spectrometry (MS) (Table 1). The selection of the bands was based upon the intensity of the band on the silver stained gel, as well as the immuno-reactivity observed after immunoblotting with horse serum (Figure 1B) or L2-mice serum (Figure 1C). Three proteins in 4 out of the 14 selected bands gave a significant score (p < 0.05) and were identified as actin (Figure 1A, band 8), glyceraldehyde 3-phosphate dehydrogenase (GAPDH) (Figure 1A, band 9) and hemoglobin (Figure 1A, bands 13 and 14). A different migration pattern was observed for the LCE3 on the 1-D silver-stained gel (Figure 1D). Analogously, immunoblots were performed with horse sera (Figure 1E) and sera of L3-mice (Figure 1F). The selection of 13 bands, indicated with arrows (Figure 1D), was based upon the same criteria as above. They were isolated for further identification by MS (Table 2). Ten proteins out of the 13 selected bands gave a significant score (p < 0.05) and were identified as the alpha chain of larval serum protein (Figure 1D, bands 1–4), arylphorin (Figure 1D, band 6), beta chain of larval serum protein (Figure 1D, band 7), hemoglobin (Figure 1D, bands 10–12) and murein lipoprotein (Figure 1D, band 13).

**Table 1:** Mass spectrometry identification of proteins identified from the LCE of L2.

Band ID	Protein name	Species	Accession number	MW (Da)	p*I*	Protein score
8	Protein similar to Actin-87E isoform 2	*Drosophila melanogaster*	AAM29410	37816	5.36	223
9	Glyceraldehyde-3-phosphate dehydrogenase	*Drosophila hydei*	S24630	35369	8.2	224
13	Hemoglobin	*Gasterophilus intestinalis*	O96457	17912	8.44	440
14	Hemoglobin	*Gasterophilus intestinalis*	O96457	17912	8.44	144

Spots assignments refer to Figure 1. Proteins listed have been identified with a significant probability score at p < 0.05 in MSDB.

**Table 2:** Mass spectrometry identification of proteins identified from the LCE of L3.

Band ID	Protein name	Species	Accession number	MW (Da)	p*I*	Protein score
1	Larval serum protein 1 alpha chain precursor	*Drosophila melanogaster*	LSP1A_DROME	98802	5.72	92
2	Larval serum protein 1 alpha chain precursor	*Drosophila melanogaster*	LSP1A_DROME	98802	5.72	89
3	Larval serum protein 1 alpha chain precursor	*Drosophila melanogaster*	LSP1A_DROME	98802	5.72	98
4	Larval serum protein 1 alpha chain precursor	*Drosophila melanogaster*	LSP1A_DROME	98802	5.72	102
6	Arylphorin subunit A4 precursor	*Calliphora vicina*	ARY1_CALVI	92282	5.59	71
7	Larval serum protein 1 beta chain precursor	*Drosophila melanogaster*	LSP1B_DROME	95849	5.41	69
10	Hemoglobin	*Gasterophilus intestinalis*	O96457	17912	8.44	247
11	Hemoglobin	*Gasterophilus intestinalis*	O96457	17912	8.44	132
12	Hemoglobin	*Gasterophilus intestinalis*	LPEBWM	17912	8.44	112
13	Major outer membrane lipoprotein precursor (Murein-lipoprotein)	*Pectobacterium atrosepticum*	LPP_ERWCT	8396	9.36	69

Spots assignments refer to Figure 1. Proteins listed have been identified with a significant probability score at p < 0.05 (1, 2, 3, 4, 6, 7, 13: Expasy database; 10, 11, 12: MSDB).

### 2-D analysis of the LCE of L2

**Figure 2:**
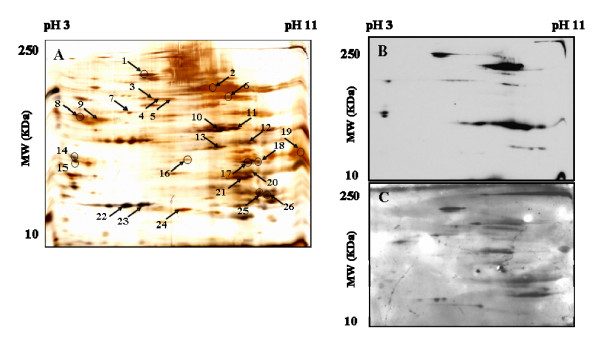
**2-D analysis of the LCE of L2**. Silver-stained representative 2-D protein map of the LCE of L2 comprising pH gradient from 3 to 11 with the MWs ranking from 10 to 250 KDa (A). The silver-stained gel was used to align detected protein spots on the Western blots performed with serum of mice immunised with the LCE of L2 (B) and with horse serum (C). The circles indicate the spots that were immunolabelled with mice serum (B; spots 1,2,6,8,14–19,25,26) and the arrows indicate the spots that were immunolabelled with horse serum (C; spots 1–13 and 16–26). A total of 26 spots were isolated for further MS identification. The protein identification by MS is presented in Table 3.

The protein migration of the LCE of L2 on a 2-D gel (Figure 2A) showed the presence of proteins all along the pH spectrum. Western blots were performed with L2-mice serum (Figure 2B) and with horse serum (Figure 2C). The silver-stained gel was used to align detected protein spots on both immunoblot profiles. A larger range of immuno-reactive proteins was observed on the horse immunoblot profile than on the L2-mice immunoblot. Circles indicate 12 spots that were considered as immuno-reactive with L2-mice serum (Figure 2A, spots: 1, 2, 6, 8, 14–20, 25) and arrows indicate 24 spots that were considered as immune reactive with horse serum (Figure 2A, spots: 1–13 and 16–26). A total of 26 spots were selected for MS analysis (Table 3). Among the 26 selected spots, 7 proteins were successfully identified (p < 0.05) by MS: paramyosin (Figure 2A, spot 1), serum albumin (Figure 2A, spot 5), tubulin (Figure 2A, spot 9), enolase (Figure 2A, spot 11), tropomyosin (Figure 2A, spot 14), GAPDH (Figure 2A, spot 19) and hemoglobin (Figure 2A, spot 20).

**Table 3:** Mass spectrometric identifications of proteins identified from the LCE of L2.

Spot ID	Protein name	Species	Accession number	MW (Da)	p*I*	Protein score
1	Paramyosin	*Drosophila melanogaster*	S22028	102277	5.5	97
5	Serum albumin precursor	*Bos taurus*	ABBOS	69225	5.8	99
9	Tubulin alpha-1 chain	*Drosophila melanogaster*	A26488	49876	5.0	260
11	Enolase	*Oryza sativa*	Q7XBE4	47942	5.4	80
14	Tropomyosin	*Drosophila melanogaster*	C25242	32740	4.7	78
19	Glyceraldehyde-3-phosphate dehydrogenase	*Drosophila hydei*	S24630	35369	8.2	100
20	Hemoglobin	*Gasterophilus intestinalis*	O96457	17912	8.4	209

Spots assignments refer to Figure 2. Proteins listed have been identified with a significant probability score at p < 0.05 in MSDB.

### 2-D analysis of the LCE of L3

**Figure 3:**
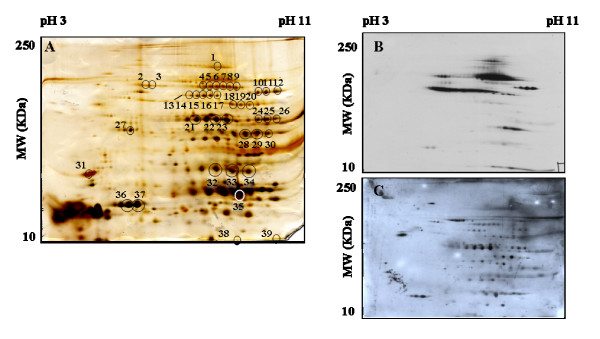
**2-D analysis of the LCE of L3**. Silver-stained representative 2-D protein map of the LCE of L3 comprising pH gradient from 3 to 11 with the MWs ranking from 10 to 250 KDa (A). The silver-stained gel was used to align detected protein spots on the Western blots performed with serum of mice immunised with the LCE of L3 (B) and with horse serum (C). 39 spots immunolabelled with both sera were isolated for further MS identification. The protein identification by MS is presented in Table 4.

The proteomic profile of the LCE of L3 presented on figure 3A shows that most of the proteins are located in a basidic range between pH 7–11. Western blots were performed with L3-mice serum (Figure 3B) and with horse serum (Figure 3C). The intensity of the immune reaction differs when the LCE of L3 is exposed with L3-mice serum or with horse serum but the immunoblot profiles were similar. After comparison of the immunoblots with the silver-stained gel, 39 spots, indicated by circles were selected for further MS identification (Table 4). 19 out of the 39 isolated spots were successfully identified (p < 0.05), corresponding to 8 different proteins: filamin (Figure 3A, spot 1), heat shock protein (HSP-70) (Figure 3A, spot 2), serum albumin precursor (Figure 3A, spot 3,18–20), phosphoenolpyruvate carboxykinase (PEPCK) (Figure 3A, spot 8), enolase (Figure 3A, spot 22,23), fumarase (Figure 3A, spot 1), beta-actin (Figure 3A, spot 27), hemoglobin (Figure 3A, spot 32–39).

**Table 4:** Mass spectrometric identifications of proteins identified from the LCE of L3.

Spot ID	Protein name	Species	Accession number	MW (Da)	p*I*	Protein score
1	Filamin 1	*Drosophila melanogaster*	Q8T3K7	151931	5.72	76
2	Heat shock 70 kDa protein 70C	*Drosophila melanogaster*	HSP7A_DROME	70871	5.34	70
3	serum albumin	*Bos taurus*	AAN17824	71274	5.82	171
8	Phosphoenolpyruvate carboxykinase	*Drosophila melanogaster*	PPCK_DROME	71882	6.07	79
18	Serum albumin precursor	*Bos taurus*	ALBU_BOVIN	71244	5.82	108
19	Serum albumin precursor	*Bos taurus*	ALBU_BOVIN	71244	5.82	117
20	Serum albumin precursor	*Bos taurus*	ALBU_BOVIN	71244	5.82	165
22	Enolase (Fragment)	*Drosophila subobscura*	O44101	44548	5.92	142
23	Enolase	*Schistosoma mansoni*	ENO_SCHMA	47421	6.18	163
24	Fumarase	*Drosophila melanogaster*	Q9VTI5	51239	8.47	111
27	Beta-actin	*Danio rerio*	ACTB1_BRARE	42082	5.3	184
32	Hemoglobin	*Gasterophilus intestinalis*	O96457	18026	8.44	95
33	Hemoglobin	*Gasterophilus intestinalis*	O96457	18026	8.44	113
34	Hemoglobin	*Gasterophilus intestinalis*	O96457	18026	8.44	131
38	Hemoglobin	*Gasterophilus intestinalis*	O96457	18026	8.44	415
39	Hemoglobin	*Gasterophilus intestinalis*	O96457	18026	8.44	410

Spots assignments refer to Figure 3. Proteins listed have been identified with a significant probability score at p < 0.05 (2, 8, 18, 19, 20, 23, 27: Expasy database; 1, 3, 22, 24, 32, 33, 34, 38, 39: MSDB).

## Discussion

The comparison of the migration patterns of the LCE2 and LCE3 in both dimensional analysis (1-D and 2-D), indicates that the larval proteinic profile is stage specific, thus suggesting a different composition in larval metabolism and antigenic properties. The 1-D silver-stained gels of the larval crude extracts indicated a very dense concentration of proteins; consequently the alignment of the immuno-reactive bands detected by horse and mice might present some differences. The proteomic approach confirmed the specific protein profile of both larval stages and allowed better identification of the different spots.

Mice were artificially immunised against a crude protein extract from whole larvae. A large number of proteins that are normally not directly in contact with horses were presented to the immune system of the mice. Furthermore, unlike natural infection eliciting a horse immune reaction, subcutaneous injection in mice induces a systemic immune reaction shifted to a Th1 response by the adjuvant. This difference in immune reaction explains the fact that most of the L2 and L3 proteins that reacted with mice sera showed a more intense signal when compared with the reaction with horse sera. Since the lifecycle of *G. intestinalis *occurs in the gastro-intestinal tract of horses, the mucosal immune system is in contact with the larvae and thus exposed to excreted or secreted substances during larval migration and development [18]. Although L2 migration patterns from mouth to stomach remains unclear, the immune reaction detected in horses and in experimentally immunized mice might suggest that the larval stage L2 possesses more antigenic proteins than the larval stage L3, probably useful for larval enzymatic migration [19]. Accordingly, enolase was identified by MS and has previously been reported as an important enzyme localized on the surface of several pathogens when invading tissue [20]. L2 is a stage inducing a strong host immune response so that its development into L3 in the stomach might be a defence mechanism of the larvae to bypass the horse immune defences. Conversely, the fact that L3 remains attached for 8–10 months to the stomach wall suggests a hypometabolic status, or reduced immunogenic properties. This can explain the weak immune reaction observed in the presence of horse serum against the L3.

Two important larval proteins, arylphorin and LSP-2 (larval serum protein), respectively homologous to *Calliphora vicina *and *Drosophila melanogaster*, were identified in the L3. In holometabolous insects the construction of adult tissues during metamorphosis requires a large amount of energy. It is known that before formation of the puparium, the fat body cells reabsorb proteins and other macromolecules that have accumulated in the haemolymph during the larval feeding period [21]. The major fraction of incorporated proteins consists of arylphorins and LSP-2 [22]. In addition, hemoglobin was identified in both larval stages of *G. intestinalis*. This abundant and circulating molecule is present in highly tracheated cells forming the posterior spiracular plate and allows the larvae to make better use of intermittent contact when air is swallowed with food [23].

Most of the other identified proteins in L2 (paramyosin, tubulin, tropomyosin, GAPDH and a protein similar to actin) or in L3 (filamin, fumarase, PEPCK, HSP-70, enolase) are shared with those of *Drosophila *spp. suggesting that structural or metabolic homologies do exist between these species.

During this research, different intestinal parasites (*Anoplocephala perfoliata*, *Parascaris equorum*, Cyathostominae) were simultaneously present with *G. intestinalis *in the gastro-intestinal tract of the slaughtered horses. The risk of cross-reactivity has still to be evaluated. But unlike some immunological studies about intestinal helminths in equids [24-26], the cross-reactivity between *G. intestinalis *and gastro-intestinal parasites has not yet been studied.

This work provides further information into the understanding of the interaction between *G. intestinalis *and their host and by contributing a novel scheme of the proteomic profile of the main larval stages. Thus our results further demonstrate the complexity of this host-parasite interaction. Indeed, this study reveals the necessity to develop a reliable serological tool to detect infested horses, particularly because the only means to detect a *G. intestinalis *infestation is by necropsy.

The identification of most of the proteins will be the next step to define their role, their cellular or tissue localisation and their potential antigenic properties.

## Methods

### Larval collection and antigen preparation

*Gasterophilus *spp. L2 and L3 were collected from the pyloric portion of the stomach of horses originating from two farms located in the District of the Swiss Jura; Delémont: N 47°21'; E 7°20', Switzerland. Simultaneously, the gastro-intestinal tract of each animal was examined and the presence of *P. equorum*, *A. perfoliata *and Cyathostominae was observed in any case.

All the larvae collected were washed in a sterile phosphate saline buffer (PBS 0.1 M, pH 7.2), identified as *G. intestinalis *on the basis of morphological keys [1] and frozen at -20°C.

For preparation of the larval crude extracts, 10 L2 larvae harvested on two different horses of a same herd, and seven L3 larvae harvested on one horse originating from the second herd, were sonicated and homogenised on ice under sterile conditions. The homogenate was extracted overnight in a 0.1 M pH 9.6 carbonate buffer containing 1 mM phenylmethylsulfonyl fluorid (PMSF) and 5 mM ethylenediamin tetraacetic acid (EDTA) by further adding 1 ml/gr of a protease inhibitor (Sigma-Aldrich, Buchs, Switzerland). The extracts were centrifuged at 20'000 × *g *for 30 minutes (4°C). The supernatant containing the antigens was collected and the final protein content determined by spectrometry (Bradford method, BioRad). LCE was lyophilized and stored at -20°C.

### Horse serum samples

Blood samples were taken on a group of twenty horses originating from the two farms in the above described area. Although blood samples and larval collection could not be made on the same animals our observations made during a simultaneously performed epidemiological survey (Roelfstra et al, in prep.), including field observations on live animals and in slaughterhouses, confirmed the high level of endemicity of *G. intestinalis *previously described by Brocard [19] in the same area and allow us to presume that all horses used in this study are infested by *G. intestinalis*. Foetal horse serum was used as a negative control for the Western blots. The blood was centrifuged at 3500 × *g *for 15 minutes at room temperature and the sera were stored at -20°C.

### Immunisation of mice and serum samples

Balb/c mice were immunised with LCE of L2 (L2 mice) or with LCE of L3 (L3 mice) from *G. intestinalis*. Two grams of L3 and two grams of L2 were sonicated, homogenised in a sterile PBS buffer pH 7.2 and centrifuged at 20'000 × *g*. The supernatant was then emulsified in equal volume of Freund's Incomplete Adjuvant (Sigma-Aldrich, Buchs, Switzerland).

A dose of 100 μg protein/mouse, in a volume of 200 μl, was injected by intramuscular route every 2 weeks through 6 weeks. Animals were bled seven weeks after the first immunisation. Control mice were inoculated with a combination of PBS and Freund's Incomplete Adjuvant every time above. Sampled blood was centrifuged at 3500 × *g *for 15 minutes at room temperature and sera stored at -20°C. All procedures were approved by the responsible local committee on animal experimentations.

### One dimensional electrophoresis (1-D)

The proteins of L2 (5 μg/well) and L3 (5 μg/well) were separated on gradient SDS-PAGE gels (4–20%) under reducing conditions (2-β-mercaptoethanol, 95°C for 5 min). Electrophoresis was performed at 80/100 V for 30 min/2 hrs. One set of the gels was stained with silver for mass spectrometry, and the second was transferred onto nitrocellulose membranes (GE Healthcare, Uppsala, Sweden) for Western blot analysis.

### Two dimensional electrophoresis (2-D)

Lyophilized LCE samples were solubilised in 2-DE lysis buffer (9 M urea, 2 M thiourea, 1% dithioerythriol, 4% CHAPS, 2.5 μM EGTA, and 2.5 μM EDTA). Immobiline dry strips pH 3–11 non linear, 11 cm (GE Healthcare, Uppsala, Sweden) were immersed overnight in lysis buffer containing 75 μg protein sample, additional 1% Pharmalyte pH 3–10 (GE Healthcare, Uppsala, Sweden), and 0.5% bromphenol blue. IEF on a Multiphor (GE Healthcare) for 15 kV·h at 20°C was followed by separation on gradient SDS-PAGE gels (9–15%) at constant 45 V per gel. One set of gels was stained with silver for MS and the second was transferred onto nitrocellulose membranes (GE Healthcare, Uppsala, Sweden) for Western blot analysis.

### Western blot analysis (WB)

Non-specific binding was blocked with 1% polyvinylpyrrolidone in PBS-Tween for 1 hour. Blots were subsequently incubated with primary antibody in PBS-Tween (overnight at 4°C; horse sera or mice sera 1:1000) and washed. The immunoreactive spots were detected using a goat anti-mouse IgG (H+L) (1:20000, Nordic Immunology Laboratories, Tilburg, The Netherlands) or an anti-horse IgG (1:10000, Sigma-Aldrich, Buchs, Switzerland) antibody conjugated with horseradish peroxidase. Signals were detected with ECL (enhanced chemiluminescence) on Hyperfilm ECL (GE Healthcare, Uppsala, Sweden) [27]. After ECL detection, the blots were subsequently stained with colloidal gold in order to match the visible spots to overall pattern on silver-stained gels.

### Mass spectrometry (MS)

Selected spots were excised from 2-D gels, destained, processed by proteolysis with trypsin [28] and analyzed by MALDI-TOF and MS/MS on a MALDI-TOF/TOF tandem mass spectrometer (ABI 4700 Proteomics Analyzer, Applied Biosystems). Combined PMF (peptide mass fingerprint) and MS/MS queries were done with MASCOT^© ^Database search engine v1.9 [29] (Matrix Science) embedded into GPS-Explorer Software (version 3.6, Applied Biosystem) on the Swiss-Prot database (version 20051206; 201594 sequences; 73123101 residues) or MSDB (version 20040703; 1501893 sequences; 480537664 residues). Protein identification was considered positive (Tables 1, 2, 3 and 4) if (i) the probability-based MOWSE score [30] obtained from both MS and MS/MS analysis was significant (i.e. scores > 66 were significant at p < 0.05 for Expasy database, and scores > 74 significant at p < 0.05 for MSDB database; confidence interval > 99% as given by GPS explorer, version 3.6); (ii) the matched peptide masses were abundant in the spectrum; and (iii) the theoretical molecular weights (MW) of the significant hits fit the experimental observed values.

## Competing interests

The authors declare that they have no competing interests.

## Authors' contributions

LR and KP conceived and designed the study. LR carried out the acquisition of the material and the morphological data as well as the immunoassays and drafted the manuscript. KP supervised the work and helped to write the manuscript. CD outlined and supervised all the proteomic features and helped to draft the manuscript. SMH carried out the mass spectrometry and analysed the data. CB has supported LR in all the laboratory analysis. MM conceived and carried out the immunological study with mice. BB participated in the design and coordination of the study and helped to analyse the results. All authors read and approved the final manuscript.
